# Is serum BDNF level relıable parameter ın detectıng of dental anxıety before ımpacted thırd molar surgery?

**DOI:** 10.4317/medoral.26558

**Published:** 2024-06-22

**Authors:** Efe Can Sıvrıkaya, Onur Yılmaz, Tamer Tuzuner, Yavuz Tolga Korkmaz, Ahmet Alver, Sefa Merve Arıkan, Nejdet Kocak, Elıf Sahın

**Affiliations:** 1 Department of Oral Maxillofacial Surgery, Faculty of Dentistry, Karadeniz Technical University, Turkey; 2 Department of Pedodontics, Faculty of Dentistry, Karadeniz Technical University, Turkey; 3 Department of Medical Biochemistry, Faculty of Medicine, Karadeniz Technical University, Turkey; 4 Bayburt Public Dental Hospital, Turkey; 5 Antalya Public Dental Hospital, Turkey

## Abstract

**Background:**

Brain-derived neurotrophic factor (BDNF) is a factor that implicate in the pathophysiology and treatment of depression and anxiety. The aim of this study was to determine the relationship between dental anxiety and BDNF serum level through impacted third molar surgery.

**Material and Methods:**

In this randomized, double-blind, cross-sectional study, the sample included patients who had been admitted for the impacted third molar extraction under local anesthesia between January to November 2020. The primary predictor variable was serum BDNF level and the second predictor variable was dental anxiety scores before and after operation in patients. The primary outcome variable was the correlation between anxiety scores (APAIS, MDAS, STAI, VAS) and serum BDNF level. The sample included 55 patients (22 Male, 33 Female) aged 18 to 42 (24,2+5,55).

**Results:**

Comparison of pre-operative scores (APAIS, MDAS, STAI, VAS and BDNF) and post-operative scores were statistically significant (*P* < .05). Post-operatively, MDAS and VAS scores decreased, while BDNF levels and STAI scores increased compared to the preoperative scores. BDNF was not correlated with APAIS, MDAS, STAI, and VAS preoperatively and postoperatively.

**Conclusions:**

There may be a relationship between serum BDNF level and dental anxiety scale, but, no correlation was found between them.

** Key words:**BDNF, dental anxiety, dental extraction, ımpacted molar surgery.

## Introduction

Dental anxiety is a term used to describe fear, anxiety or stress during or before the dental practice. Many patients feel anxious before dental treatment on the presumption that the procedure will cause them pain and discomfort (1). Increased anxiety levels can lead to systemic distress such as increased blood pressure and vagal reflex (2), impaired cognitive feelings (3), impaired surgical performance and increased operating time (4). Surgical procedures such as extraction of impacted teeth have been reported to increase dental anxiety and fear more than other dental treatments (5). Information (6), music (7), hypnosis (8), medicines as midazolam (9), virtual reality (7) have been suggested to reduce dental anxiety but it cannot be eliminated completely. Therefore, the detection of dental anxiety before dental treatment could be useful in preventing complications (1).

There are many dental anxiety scales (2,10) about dental practice in the literature for determining the level of dental anxiety. These scales are preferred because they are non-invasive and easy to apply but have disadvantages such as decreased reliability due to patient non-compliance, increased length of stay in the dental chair, and difficulties in applying it to patients. Today, anxiety measurement assessments are available with salivary cortisol, salivary alpha amylase and serum levels of D vit with dental anxiety methods from saliva and blood fluid (11). However, there is still controversy in the literature regarding the use of these markers in dental anxiety because they are not only related to anxiety. Cortisol, for example, is also increased by acute fear (11). Vitamin D is often associated with nutrition (12). It has also been reported that in saliva analysis such as alpha amylase or cortisol, many factors such as the region, time of sampling and infection may change the result so evaluation of serum scores may be more accurate (13).

In recent years, there has been growing evidence to show that the brain-derived neurotrophic factor (BDNF)/TrKB pathway can modulate serotonin (5-hydroxytryptamine; 5-HT) and other neurotransmitters, and this pathway has been implicated in the pathophysiology and treatment of depression and anxiety (14). BDNF is released by astrocyte and neural cells in the hippocampus and generally uses a dual receptor system - it preferentially binds to the TrkB receptor but also binds to a low-affinity receptor, p75 neurotrophin receptor (p75 NTR) and regulates the release of many neurotransmitters. These are involved in the maintenance of cell plasticity, growth and death (apoptosis) also ensure the maintenance of synaptic connection (15). Clinical data have also revealed epigenetic changes in the BDNF gene in adult male patients with anxiety and depression (14,16) and increases in BDNF levels following treatment with fluoxetine, an antidepression drug (14,16). Thus, BDNF level in the brain can be a potential therapeutic means for detecting of dental anxiety

In present study, dental anxiety scales -Amsterdam Preoperative Anxiety and Information Scale (APAIS), Modified Dental Anxiety Scale (MDAS) and Spielberger’s State-Trait Anxiety Inventory (STAI), VAS- and BDNF serum levels were recorded before and after the operation in patients who had impacted tooth extraction. The aim of this study was to determine the correlation between dental anxiety and BDNF serum levels before and/or after surgery and to describe whether BDNF can be used as a marker for the detection of dental anxiety through impacted third molar surgery. The investigators hypothesize that dental anxiety is correlated with BDNF serum levels.

Materıal and Methods

- Study desıgn/sample

The randomized, prospective study included a total of 55 patients aged 18 to 42 scheduled to undergo impacted lower third molar removal at the Department of Oral and Maxillofacial Surgery Karadeniz Technical University, Faculty of Medicine, Scientific Ethical Committee from January to November 2020.

The institutional review board of Karadeniz Technical University approved the present study (approval no. 24237859-629; September 13, 2019) in accordance with the principles of the Declaration of Helsinki and the recommendations of the CONSORT (consolidated standards of reporting trials) guidelines. All patients involved signed an informed consent agreement describing the procedures and the objectives of the study prior to their inclusion in the research.

Inclusion criteria of the present study; systemically healthy (ASA-1), indication for extraction of asymptomatic mandibular third molars of class IIB according to tooth classification of Pell and Gregory (1933), mesioangular position of the tooth. The exclusion criteria were as follows: psychiatric illness or systemic disease, refusal to participate present study, incomplete forms, incomplete data available, previous unattractive dental treatment experience, a history of anxiety attacks and/or anxiolytic treatment, an inability to cooperate, tobacco usage, and pregnancy or lactation.

Patients that met the criteria to participate in the research were selected according to the order of admission to the clinic randomly.

- Study varıables

The primary predictor variable was serum BDNF level and the second predictor variable was anxiety scores before and after operation in patients. Blood samples (5 ml each time) were taken from patients in the operating room 1 hour before and immediately after surgery (total 10 ml). Serum was separated with a centrifuge at 3,000 rpm and 4°C for 10 min and stored at −80 °C till further analysis. Serum BDNF levels were measured from blood serum samples by using the commercial ELISA kit (Boster, Pleasanton California; Cat Nr: EK0307). The basis of the kit was based on the sandwich-ELISA detection method. After the procedure steps were followed in line with the manufacturer’s recommendations, absorbance was detected at 450 nm wavelength in the microplate reader (VERSA Max Molecular Devices). The results were calculated with the aid of a standard graph and expressed as pg/mL.

**Figure 1:**
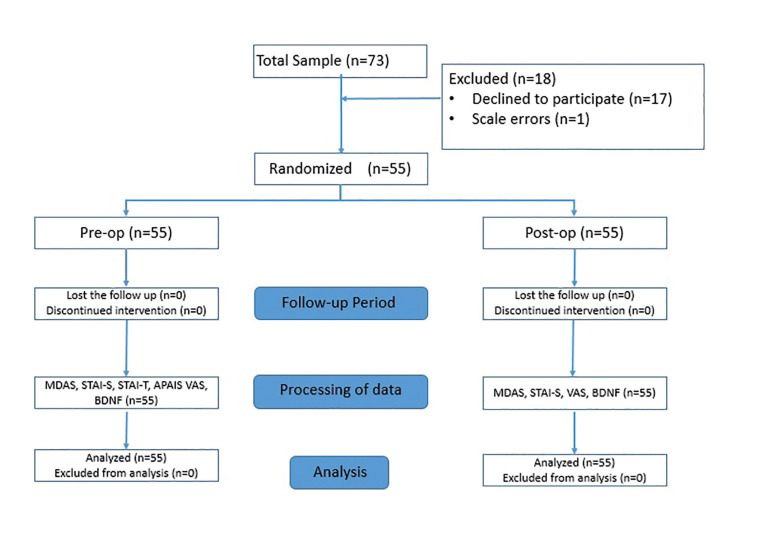
Patient flow diagram.

APAIS, MDAS, STAI and VAS were applied to the patients 1 hour before the surgical operation. Immediately after surgery, MDAS, STAI and VAS were applied to the patients. The serum BDNF level of the patients was measured 2 times from each blood sample with the Elisa kit (Boster, Pleasanton, California) before and after the surgery (Fig. 1). In addition, the participant’s age and gender were queried.

The primary outcome variable was the correlation between anxiety scores and serum BDNF levels. The secondary outcome variable were anxiety scores and BDNF changes before and after the operation in patients.

- Questıonnaıres

In the present study, the STAI-T, STAI-S, MDAS, APAIS and VAS scales, which provide useful information about patient anxiety and have a positive correlation, were used for a comprehensive assessment of patient anxiety.

STAI-S, like kinetic energy, refers to a palpable reaction or process taking place at a given time and level of intensity. STAI-T, like potential energy, refers to individual differences in reactions. Both of STAI-S and STAI-T scales include 20 questions. For each question, the scores range from 1 (almost never) to 4 (almost always) points. For both scales, the sum of the scores ranges from 20 to 80 (6).

The MDAS questionnaire is a modification of Corah’s Dental Anxiety Scale and consists of 5 questions that measure anxiety at different stages of dental treatment. The options range from 1 (not anxious) to 5 (very anxious) for each question. The sum of the scores varies from 5 to 25 (17,18).

The APAIS consists of 6 items, 3 related to anesthesia and 3 related to the surgical procedure. The total score ranges from 6 to 30 (10).

The VAS scale was included in present study in order to determine how anxious the patients feel with their thoughts with the score ranging from 0 (no anxiety) to 10 (extreme anxiety) (19).

- Data collectıon methods

Data on the age and gender of patients and the preoperative and postoperative variables (anxiety scores and serum BDNF level) were collected. Anxiety scores (APAIS, MDAS, STAI and VAS) were calculated independently by two maxillofacial surgeons. Non-identical scores were recalculated by them and a final decision was made to reach a statistical analysis. Also, BDNF was calculated using a standardized graph and expressed in pg/mL by two biochemistry experts. The study was completely blinded because the investigators who determined the BDNF results and anxiety scores were different and the patients were numerically named. All impacted third molars surgeries were performed by a single oral surgeon under local anesthesia in the morning times (8.00 am-10.00 am). The surgeon used an envelope flap design following regional anesthesia. The impacted tooth in the mesioangular position (class 2b Pell and Gregory) was completely removed by cutting with a conventional burs. The flap was fixed primary with a silk suture.

Post-op recommendations were given to all patients. All patients were prescribed routine postoperative medications, including antibiotics (amoxicillin 500 mg 2 times a day), analgesics (paracetamol 500 mg 3 times a day), and mouthwash (2% chlorhexidine gluconate 3 times a day). The sutures were removed on the 7th day after the surgery.

- Data analysıs

The sample size was calculated by using G-Power software version 3.1.2 (FF, Universitat Kiel, Germany). The alpha and beta levels were selected as 0.05 and 0.01, the effect size was determined as 0.6 according to the previous findings (20). The required sample size was determined as 48 for the study population. However, by considering the potential data losses, the final sample size was obtained as at least 55 patients.

Statistical analyses were performed with SPSS for Windows 17.0 (SPSS Inc. Chicago, IL, USA). The normality of data was tested by using Kolmogorov Smirnov test. The descriptive values were given as frequencies, mean, standard deviation, median, minimum and maximum. The comparisons were done by using Mann Whitney U and Wilcoxon signed rank tests. The Spearman correlation test was also used. *p*<0.05 was considered as significant.

## Results

A total of 73 patients who met the criteria to participate were included in present clinical study (Fig. 1). Patients who did not fill the scales completely (*n*=1) and did not approve post-procedure blood tests (*n*=17) were excluded from the study. Thus, a total of 55 patients [33 female (60%) and 22 male (40%); mean age, 24,2 ±5,5 and median age 22 (18-42) years] were included in the present study. The right or left mandibular impacted third molar was extracted in each patient. The comparison of anxiety scales (MDAS, STAI-s, VAS) and serum BDNF levels between genders was given in Table 1 and there were no statistical differences (*P* > .05).

The MDAS, STAI-S, VAS and BDNF scores at the pre-operatively and post-operatively are listed in Table 2. The comparisons of pre-op and post-op scores for MDAS, STAI-S, VAS and BDNF were statistically significant (*P* < .05). Post-op scores of BDNF and STAI-S increased, MDAS and VAS decreased (Table 2).

Correlations of preoperative and postoperative scores of MDAS, STAI-S, VAS and BDNF are shown in Table 3. Preoperatively, STAI-S-MDAS and MDAS-VAS scores were correlated (*P* < .05; Table 3). Postoperatively, STAI-S, MDAS, and VAS were correlated (*P* < .05; Table 3). Pre-op MDAS; was correlated with post-op VAS and post-op MDAS (*P* < .05; Table 3). Pre-op STAI-S was correlated with post-op MDAS and post-op STAI-S (*P* < .05; Table 3). Pre-op VAS was correlated with post-op MDAS and STAI-S (*P* < .05; Table 3). Post-op MDAS was correlated with Pre-op VAS and Pre-op STAI-S. Post-op STAI-S was correlated with Pre-op VAS (*P* < .05; Table 3). Post-op VAS; was correlated with pre-op MDAS and pre-op VAS (*P* < .05; Table 3). BDNF was not correlated with other scales preoperatively and postoperatively (*P* > .05; Table 3). Pre-op BDNF was correlated with Post-op BDNF (*P* < .05; Table 3). The STAI-T and APAIS scales were not correlated with pre-op BDNF and Post-op BDNF (*P* > .05; Table 4). The STAI correlations were negative and the other correlations (MDAS, VAS and BDNF) were positive in Table 3 and Table 4.

Dıscussıon

There are studies stating that increases in BDNF serum level has stress-reducing (20), antidepressant effects (21) and development of mood disorders (15), but the relationship between dental anxiety and BDNF has not been described in the literature. In present study, the use of serum BDNF level in the determination of dental anxiety through third molar surgery was evaluated. The aim of the study was to determine the patient's pre-procedural dental anxiety through the serum BDNF level detected with Elisa kits during the initial examination. The correlation between dental anxiety scales (MDAS, STAI, and APAIS), VAS and BDF serum concentration levels were evaluated. There was a statistical difference in pre-operative and post-operative values in anxiety scales, VAS and BDNF (*P* < .05) but there was no correlation between pre- and postoperative BDNF and APAIS, MDAS, STAI, VAS (*P* > .05). The hypothesis of a correlation between dental anxiety scale scores and serum BDNF level was rejected.

Studies on postmortem human materials stated that the hippocampus of patients with depression has lower levels of BDNF, BDNF-regulated genes, and TrkB (22). Domenica Sanna *et al*. (23) stated that BDNF, GAP-43 and p-NFH protein increase are linked events required for enhanced regeneration after nerve injury. Similarly, Sanna *et al*. (23) described that protein increase are linked events required for enhanced regeneration after nerve injury. Also, BDNF signaling is described that has been implicated as anti-depressant and anxiolytic effects (15,21). There are no studies in the literature investigating the relationship between dental anxiety and BDNF. Rael Cahn (20) stated that there was a significant positive correlation between improvements in depression and increases in BDNF in individuals who practice yoga. Also, it is stated that antidepressant drugs increase BDNF expression within the hippocampus (21). Also, this mechanism becomes active with an increase in anxiety. Saruta and al. (21) described in their study in mice that the mice characteristically show increased blood BDNF and anxiolytic behavior. Similarly, it was observed that BDNF increased statistically different after the operation in present study. It is considered that BDNF is increased due to postoperative stress.

In present study, according to MDAS and VAS scores, anxiety decreased after the operation. After surgery, it is normal to feel less anxious as the uncertainty and concern of the surgery is over. Appukuttan *et al*. (19). described that there was a negative relationship between dental anxiety and serum BDNF level and the VAS used in present study was compatible with MDAS similar to this study. However, according to STAI-S scores, post-operative anxiety increased. Gomez *et al*. (24) described in their study a negative relationship between the STAI and MDAS scales. Similarly, post-operative BDNF and STAI-S increased, MDAS and VAS decreased in the present study. Also, there are studies stated that STAI and MDAS are positive relationship when assessing dental anxiety in the literature (25). The reason for the inconsistency between the scales may be related to the usage areas (6,10,17,18). While MDAS is an anxiety scale applied for dentistry (17,18), STA-I is a general condition assessment scale (6). According to Table 3, there is no advantage of any dental anxiety scale over the other in terms of its correlation with BDNF. However, since dental anxiety scales, we recommend investigating the correlation of the VAS and MDAS scales with BDNF in future studies.

Several reports have documented BDNF concentrations in human serum /plasma but there were conflicting results regarding them (26). In present study, there was no correlation between BDNF and anxiety scales. It may be that the rise and fall of anxiety is situational (6,17-19) or general (6,10) but the subsequent rise-time trend of serum BDNF is still unclear. In present study, BDNF measurement was performed immediately after the operation. If the measurement was made after a few hours, different serum levels might be detected (26) (an increase in BDNF would have been predicted). Also, BDNF serum level can change in humans depending on age, stress, physical activity, energy, and addictions like smoking (26). Piccini *et al*. (27) described that serum/plasma concentrations of BDNF between males and females were not statistically significant similar to present study (Table 1). However, post-operatively, the tendency to decrease in MDAS scores and increase in serum BDNF levels were higher in males (Table 1). Lommatzsch *et al*. (28) failed to confirm a menstrual cycle-dependent variation of BDNF. This tendency may be due to the cognitive and affective differences between the genders.

There are saliva analyses of BDNF in the literature (11,21,29). Mandel *et al*. (29) assessed the physiological significance of human salivary BDNF and there was no relationship. In the future, it would be clinically very comforTable to detect dental anxiety from BDNF saliva analysis but, many factors such as the area where the saliva sample was taken, salivary flow-rate and timing to sample collection, stress, and infection affect the results (13). Therefore, it was decided to measure serum BDNF levels in present study. Saruta *et al*. (13) described that BDNF was consistently localized in human submandibular gland serous and ductal cells. Mandel *et al*. (29) reported that women had significantly higher levels of salivary BDNF than men. In their study, all samples were collected between 12 and 1 p.m. In addition, Saruta *et al*. (13) collected between 9 and 10 a.m and reported that there was no difference in BDNF saliva concentrations between males and females similar to present study (Table 1). The timing to sample collection was also standardized ( 8.00 a.m. - 10.00 a.m.) in present study. This period was chosen to minimize the possible effects of daily variation (13).

The present study had some limitations. Although a comprehensive assessment of patient anxiety was obtained by use of the anxiety scales, these scales provide a subjective measurement and not an objective evaluation. Secondly, this was a short-term study and the sample size in the study was limited. Studies with a large number of participants and/or time may result in different correlative values. Also, there were many variables (such as age, stress, physical activity, energy and addictions) in the sample minimizing the variables in future studies will shed light on the relationship between BDNF and dental anxiety in the literature.

In conclusion, the difference in pre-and postoperative BDNF levels suggests that it is associated with anxiety. However, it was not yet appropriate to use it as a reliable parameter in detecting dental anxiety because there was no correlation.

## Figures and Tables

**Table 1:** Comparison of genders in terms of anxiety and BDNF.

		Pre-op				Post-op			
		MDAS	STAI-S	VAS	BDNF	MDAS	STAI-S	VAS	BDNF
Male	Mean±SD	9±2,8	43,77 ±5,02	3±2,54	4102,18 ±1697,81	8,04±2,8	45,18 ±4,94	2,59 ±2,1	5426,37 ±1359,16
	Median(*)	9 (5-14)	45 (32-51)	2 (0-8)	4184,58 (1190,92-7455,44)	8 (5-17)	46,5 (31-53)	3 (0-7)	5675,51 (2832,88-8207,46)
Female	Mean±SD	9,57 ±4,27	42,51±7,08	3,33 ±2,95	4812,95 ±1781,56	9,36 ±3,94	45,63 ±8,63	2,48 ±2,58	5564,46 ±1259,76
	Median(*)	9 (5-20)	43 (25-53)	3 (0-10)	5097,09 (1137,02-8168,76)	9 (5-21)	45 (29-82)	1 (0-10)	5337,73 (3340,11-8816,97)
	z	-0,09	-0,57	-0,26	-1,39	-1,27	-0,5	-0,44	-0,11
	*P value*	0,9	0,56	0,78	0,16	0,2	0,61	0,56	0,9

(*) Min-max, Mann-Whitney U test.

**Table 2:** Preoperative and postoperative comparison of instant scales and BDNF.

		MDAS	STAI-S	VAS	BDNF
Pre-op	Mean±SD	9,36±3,73	43,01±6,32	3,2±2,77	4536,53±1768,17
	Median (min-max)	9 (5-22)	44 (25-53)	2 (0-10)	4678,01 (1137-8168,7)
Post-op	Mean±SD	8,83±3,56	45,45±7,32	2,52±2,4	5510,76±1288,37
	Median (min-max)	8 (5-21)	45 (29-82)	2 (0-10)	5347,17 (2832,8-8816,9)
z		-2,040	-2,214	-2,603	-3,147
*P value*		0,021	0,027	0,009	0,002

Wilcoxon signed rank test.

**Table 3:** Correlations of instant scales and BDNF.

		Pre-op				Post-op			
		MDAS	STAI-S	VAS	BDNF	MDAS	STAI-S	VAS	BDNF
Pre-op	MDAS	r=1	r=-0,310* *p=*0,21	r=0,513** *p=*0	r=0,024 *p=*0,86	r=0,69** *p=*0	r=-0,16 *p=*0,24	r=0,296* *p=*0,02	r=0,04 *p=*0,72
	STAI-S	r=0,31 *p=*0,021	r=1	r=-0,168 *p=*0,22	r=-0,142 *p=*0,3	r=-0,36** *p=*0,006	r=0,36 *p=*0,007	r=-0,19 *p=*0,14	r=0,15 *p=*0,25
	VAS	r=0,512 *p=*0	r=-0,16 *p=*0,22	r=1	r=0,012 *p=*0,93	r=0,28* *p=*0,03	r=-0,32* *p=*0,01	r=0,69** *p=*0	r=-0,057 *p=*0,68
	BDNF	r=0,24 *p=*0,86	r=-0,142 *p=*0,3	r=0012 *p=*0,93	r=1	r=-0,21 *p=*0,88	r=-0,14 *p=*0,31	r=-0,22 *p=*0,87	r=0,35** *p=*0,009
Post-op	MDAS	r=0,69** *p=*0	r=-0,36** *p=*0,006	r=0,28* *p=*0,036	r=-0,02 *p=*0,88	r=1	r=-0,22 *p=*0,09	r=0,29* *p=*0,02	r=-078 *p=*0,57
	STAI-S	r=-0,16 *p=*0,24	r=0,36** *p=*0,07	r=-0,32 *p=*0,01	r=-0,14 *p=*0,31	r=-0,22 *p=*0,09	r=1	r=-0,36** *p=*0,007	r=0,05 *p=*0,69
	VAS	r=0,29* *p=*0,02	r=-0,19 *p=*0,14	r=0,69** *p=*0	r=-0,22 *p=*0,87	r=0,29* *p=*0,02	r=0,36** *p=*0,007	r=1	r=-0,14 *p=*0,29
	BDNF	r=0,04 *p=*0,72	r=0,15 *p=*0,25	r=-0,57 *p=*0,68	r=0,35 *p=*0,009	r=-0,07 *p=*0,57	r=0,05 *p=*0,69	r=-0,14 *p=*0,29	r=1

*Correlation is significant at the 0.05 level ( 2-tailed). ** Correlation is significat at the 0.01 level (2-tailed). Nonparametric spearman's rho test.

**Table 4:** Correlations of general scales and BDNF.

	STAI-T	APAIS	Pre-op BDNF	Post-op BDNF
STAI-T	r=1	r=-0,14 *p=*0,28	r=-0,08 *p=*0,53	r=-0,02 *p=*0,84
APAIS	r=-0,14 *p=*0,28	r=1	r=0,15 *p=*0,27	r=0,09 *p=*0,5
Pre-op BDNF	r=-0,08 *p=*0,53	r=0,15 *p=*0,27	r=1	r=0,35** *p=*0,009
Post-op BDNF	r=-0,027 *p=*0,84	r=0,092 *p=*0,5	r=0,35** *p=*0,009	r=1

** Correlation is significat at the 0.01 level (2-tailed). Nonparametric spearman's rho test.
